# Intravenous thrombolysis and mechanical thrombectomy in acute stroke patients on direct oral anticoagulants

**DOI:** 10.1007/s00415-024-12832-0

**Published:** 2024-12-21

**Authors:** Espen Saxhaug Kristoffersen, David Julian Seiffge, Thomas Raphael Meinel

**Affiliations:** 1 Department of Neurology, Akershus University Hospital, PO Box 1000, 1478 Lørenskog, Norway; 2 Department of General Practice, Institute of Health and Society, University of Oslo, Oslo, Norway; 3 Department of Neurology, Inselspital Bern University Hospital and University of Bern, Bern, Switzerland

**Keywords:** Intravenous thrombolysis, Thrombectomy, Direct oral anticoagulants (DOACs), Factor Xa inhibitors, Acute ischemic stroke, Intracerebral hemorrhage

## Abstract

Intravenous thrombolysis and mechanical thrombectomy reduce morbidity and improve functional outcome in ischemic stroke. However, acute recanalization therapies may increase the risk of symptomatic intracranial hemorrhage due to its effects on the brain tissue. An increasing proportion of patients with ischemic stroke are using direct oral anticoagulants (DOACs). While current international guidelines recommend against intravenous thrombolysis in patients with intake of DOACs within the last 48 h, they also highlight lack of evidence in the area. Based on these guidelines, a significant proportion of patients are consequently disqualified from intravenous thrombolysis. Measuring anticoagulant activity before intravenous thrombolysis has been suggested as a way to select patients with low risk of symptomatic intracranial hemorrhage, but uncertainty exists about feasibility, validity, availability and costs. Reversal agents have demonstrated potential in facilitating safer intravenous thrombolysis administration, though their efficacy is not established in randomized controlled trials, and logistical and cost-related barriers limit their widespread use. During the last couple of years several large cohort studies reported no significant increase in symptomatic intracranial hemorrhage among selected patients on DOACs receiving intravenous thrombolysis compared to those not on anticoagulants, even without the use of DOAC plasma levels or reversal agents. Mechanical thrombectomy appears to be generally safe in patients with recent DOAC intake. The aim of this review is to discuss the uncertainty around the safety and efficacy of intravenous thrombolysis and thrombectomy in patients with a recent intake of DOAC, summarize existing knowledge, and outline potential approaches.

## Introduction

Intravenous thrombolysis (IVT) and mechanical thrombectomy (MT) improve neurologic outcomes in selected patients with acute ischemic stroke [1, 2].

IVT with recombinant tissue plasminogen activator was approved for acute ischemic stroke by the US Food and Drug Administration (FDA) in June 1996, followed by other countries including a later approval for all member states in the European Union from the European Agency for the Evaluation of Medicinal Products (EMEA) in 2002 [3]. The FDA approval was based on the NINDS trial, which was in fact two trials; a safety phase IIB trial and a phase III efficacy trial that both excluded patients who were taking anticoagulants and had an elevated partial-thromboplastin time or pro-thrombin times > 15 s [4, 5]. These original exclusion criteria were used for the FDA label and the later EMEA approval. Thus, exclusion criteria in today’s routine thrombolysis are based on those used in the early IVT trials, but most of them have now been modified after international stroke trials, subgroup analysis of earlier trials, and observational series [6–8]. One of the few remaining absolute contraindications for IVT in International guidelines is recent intake of oral anticoagulation unless coagulation tests indicate safety [6, 7]. This recommendation is based on the presumption of an increased risk of symptomatic intracranial hemorrhage (sICH) in patients on oral anticoagulants receiving IVT [9, 10]. The reported rates of sICH in patients not using anticoagulation ranges between 3 and 5% after recanalization therapies (IVT alone, MT alone or combination of IVT and MT) depending on the definition used [1, 2, 10, 11]. The pathophysiology of intracranial hemorrhage in acute ischemic stroke is complex and likely involves multiple overlapping mechanisms [10, 12].

In the absence of any data to decide otherwise, the initial recommendation for patients on vitamin K antagonists (VKAs) was extended for patients on direct oral anticoagulants (DOACs) when DOACs became available almost 2 decades later than the NINDS trial. Thus, International guidelines recommend against IVT in patients with a recent intake (< 48 h) of DOACs unless specific coagulation tests indicate safety [6, 7].

The prevalence of atrial fibrillation, and consequently the number of individuals on long-term anticoagulation therapy, is expected to double in the coming decades [13]. DOACs have during the last 10 years emerged as the primary therapeutic option for stroke prevention in patients with non-valvular atrial fibrillation [14]. DOACs inhibit specific factors in the coagulation system, targeting either thrombin (as in the case of dabigatran) or factor Xa (as with apixaban, edoxaban, and rivaroxaban)[15]. The anticoagulant effect of DOACs starts rapidly, with peak levels reached within a few hours of ingestion of apixaban (3–4 h), dabigatran (1.5–3 h), edoxaban (1–2 h) and rivaroxaban (2–3 h). The corresponding half-lives are 12 h, 11–14 h, 10–14 h and 5–15 h meaning that the anticoagulation effect lasts only for several hours to a few days in contrast to the long-lasting inhibition of the synthesis of coagulation factors II, VII, IX and X seen with VKAs [15]. Nowadays, the use of DOACs has almost replaced the use of VKAs [16, 17]. This change is driven by feasibility (no regular coagulation activity testing, less follow-ups, fewer drug and food interactions) and the lower risk of hemorrhagic complications with DOACs [16, 17].

DOACs do not prevent all future stroke among those using these medications [18]. In addition, not all patients with a prescription of a DOAC actually take their medication, and even small reductions in adherence in patients with atrial fibrillation are associated with increased risk of stroke [19, 20]. Patients may also use DOAC for other indications [15]. Recent reports found up to 13% of all patients who had a stroke to be on anticoagulant therapy prior to stroke onset [21–23]. Since the shift from VKA to DOACs, it is estimated that one in six stroke patients otherwise eligible for IVT is on a DOAC [24]. Thus, patients on DOAC represent a substantial proportion of the overall acute ischemic stroke population. Together this means that an increasing proportion of patients with acute ischemic stroke who otherwise qualify for IVT will be denied IVT due to recent DOAC intake in future. Using oral anticoagulation is already reported to be the most common absolute contraindication for receiving IVT [25].

Today’s recommendations against IVT in patients with recent DOAC intake are mostly based on expert consensus due to the lack of evidence from randomized controlled trials [6, 7]. Data regarding safety of MT in patients on DOAC relies on observational studies [26, 27]. Thus, there exists an uncertainty about whether patients on DOACs actually can receive acute recanalization therapies for acute ischemic stroke, and especially whether IVT is safe for patients on DOACs. This review will summarize existing knowledge regarding the uncertainty around the safety and efficacy of recanalization therapies in patients with acute ischemic stroke and a recent intake of DOAC, and outline potential management of this clinical problem.

## Evidence for IVT in patients with recent DOAC intake

In the few experimental ischemic stroke studies that exist on the topic, rivaroxaban, apixaban and dabigatran did not increase the chance of hemorrhage after IVT, whereas VKA did [28–33]. There are even some preclinical data that support that thrombin inhibitors can reduce the risk of intracranial hemorrhage after IVT. Thrombin activation of the protease activated receptor 1 induces cytotoxicity, cell death, microglial activation, toxins and endothelial hyperpermeability. Reducing the thrombin activity in brain, either via direct thrombin inhibitors or indirectly by Factor Xa inhibitors may block cytotoxicity to preserve the blood–brain barrier and increase cell survival as summarized in this recent review [12].

There exists no direct evidence from clinical trials regarding the use of IVT for acute ischemic stroke in patients taking DOACs as all the major IVT trials were performed before the advent of DOACs.

Several retrospective cohort studies have collected information about the safety of IVT in selected DOAC patients over the last 10 years (Table 1). No studies have tested the efficacy of IVT in patients with a recent DOAC intake.

**Table 1:** Cohort studies of > 15 patients with recent DOAC intake receiving IVT compared to controls without IVT

Design and setting	Years	Cases	Controls	Definition DOAC intake	Definition sICH	Rate sICH % (95% CI)	Comments
Retrospective, cohort study. Nationwide based on the Austrian Stroke Unit Registry [34]	2018–2023	419	15 062	Intake < 48 h of admission	Not given	4.8 (3.0 to 7.4) vs. 2.9 (2.6 to 3.1)	No detailed clinical information No information about Idarucizumab, DOAC plasma levels or types of DOAC
Retrospective, single-center target trial based on the Bernese Stroke Registry, Switzerland [40]	2021–2023	49 Apixaban *n* = 53 Edoxaban *n* = 6 Rivaroxaban *n* = 38 Dabigatran *n* = 1 Idarucizumab = 0	49*	Intake < 48 h of admission	ECASS III and NINDS	ECASS III: 0 (NA) vs. 4 (NA) NINDS: 2 (NA) vs. 9 (NA)	DOAC plasma levels available for 46 patients in each group Included patients from the extended time window
Retrospective, propensity matched cohort study. Nationwide, based on Taiwan’s National Health Insurance Research Database [35]	2011–2020	91	364**	Active DOAC prescription in claim database	Any ICH defined by diagnose codes in claim database	9.9 (NA) vs. 7.4(NA)	No information of DOAC plasma levels or types of DOAC Patients who received Idarucizumab excluded
Retrospective, international, multicenter, cohort study from Europe, Asia, Australia, and New Zealand [24]	2008–2021	832 Apixaban *n* = 163 Edoxaban *n* = 68 Rivaroxaban *n* = 258 Dabigatran *n* = 342 other *n* = 1 Idarucizumab = 252	32 375	Intake < 48 h of admission	ECASS III	2.5 (1.6 to 3.8) vs. 4.1 (3.9 to 4.4)	225 with DOAC plasma levels
Retrospective, single-center cohort study from Japan [36]	2011–2021	40 Apixaban *n* = 16 Edoxaban *n* = 10 rivaroxaban *n* = 8 dabigatran *n* = 6 Idarucizumab = 2	753	Intake < 48 h of admission	ECASS III	2.5 (NA) vs. 2.4 (NA), p = 0.95	Alteplase 0.6 mg/kg
Retrospective, multicenter, cohort study based on the Get With The Guidelines-Stroke Registry in the US [38]	2015–2020	2207	160 831	Intake < 7 days of admission	Any ICH with a clinical deterioration attributed to ICH	3.7 (2.9 to 4.5) vs. 3.2 (3.1 to 3.3)	No information of DOAC plasma levels or types of DOAC
Retrospective, single-center cohort study from Switzerland [42]	2012–2016	18 Rivaroxaban *n* = 18	41***	Intake < 48 h of admission	ECASS II and NINDS	ECASS II: 0 (NA) vs. 0 (NA)	Inclusion based on rivaroxaban plasma level
Retrospective, multicenter, cohort study based on the Get With The Guidelines-Stroke Registry in the US [37]	2012–2015	251 Apixaban *n* = 35 Rivaroxaban *n* = 129 Dabigatran *n* = 87	41 136	Intake < 7 days of admission	Any ICH with a clinical deterioration attributed to ICH	NINDS: 4.8 (NA) vs. 3.9 (NA)	No information of DOAC plasma levels Idarucizumab not available
Retrospective, international, multicenter, cohort study from Europe [39]	Up till 2014	78 Apixaban *n* = 2 Rivaroxaban *n* = 47 Dabigatran *n* = 29	8 938	Intake < 48 h of admission	ECASS II and NINDS	ECASS II: 2.6 (NA) vs. 5.0 (NA) NINDS: 3.9 (NA) vs. 7.2 (NA)	Idarucizumab not available

Studies of only dabigatran and Idarucizumab are not included

Information and numbers extracted and calculated from the publications

*Patients with a recent DOAC intake, but not receiving IVT

**Only 364 out of 7210 controls were included due to propensity score matching

***Patients with recent rivaroxaban intake, but not receiving recanalization therapy

ECASS: European Cooperative Acute Stroke Study

NINDS: The National Institute of Neurological Disorders and Stroke rt-PA Stroke Study Group

DOAC: direct oral anticoagulants

IVT: intravenous thrombolysis

sICH: symptomatic intracranial hemorrhage

NA: data not available within the manuscript

Recent data from the Austrian Stroke Registry, after adjusting for age, baseline National Institutes of Health Stroke Scale (NIHSS), hypertension, and diabetes demonstrated that the risk of sICH was not significantly different between patients treated with IVT who had recently taken DOAC and those treated with IVT with no prior oral anticoagulation (adjusted OR 1.39; 95% CI 0.85 to 2.15) [34]. However, these findings are limited by the registry’s lack of detailed data, and missing information on the use of reversal agents or DOAC plasma levels.

In a retrospective cohort study from Taiwan, 91 patients on DOACs and 7210 patients not on anticoagulants were analyzed, with the control group reduced to 364 patients through propensity score matching [35]. The study found no increased risk of ICH after IVT in patients on DOACs (OR 1.37; 95% CI 0.62 to 3.03). This study faced limitations such as a small sample size, reliance on prescription claims and administrative codes to identify recent DOAC use and treatment-related ICH.

A Japanese retrospective single-center study included 40 patients on DOACs and 753 not on anticoagulants [36]. The study concluded that pre-stroke DOAC treatment did not increase the risk of sICH after IVT (adjusted OR 0.95; 95% CI 0.17 to 5.28). The primary analysis was adjusted for age, sex, baseline NIHSS score, and Alberta Stroke Program Early Computed Tomography Score. Limitations included a small sample size and lack of external validity since the alteplase dose used in Japan is lower than in other countries, and the Japanese guidelines for IVT in patients on DOACs are more lenient than in Europe and US [36].

Retrospective data from the US Get With The Guidelines registry also found no significant increase in the risk of sICH after IVT in patients treated with DOACs compared to those not on anticoagulants [37, 38]. These two studies did not include overlapping patients and were based on different time periods (2012–2015 and 2015–2020). It should be mentioned that practice within the registry has likely evolved over time. The later cohort likely represent a less restrictive approach with using thrombolysis with concurrent DOAC use. In this latter study, 2207 patients on DOACs and 160 831 patients not on anticoagulants were analyzed. The study indicated no significant increase in risk of sICH after IVT in patients treated with DOACs compared to those not on anticoagulants (adjusted OR 0.88; 95% CI 0.70 to 1.10) [38]. The primary analysis was adjusted for several factors including, but not restricted to known risk factors for sICH such as age, hypertension, NIHSS, blood pressure, blood glucose level, medication use. The study had several limitations including DOAC intake defined as within the last 7 days prior to admission, a tiny minority of patients with confirmed intake within the last 48 h and no validated definition of sICH. Thus, it is hard to apply these findings into everyday clinical practice in the emergency room.

An international consortium pooled data for a retrospective cohort study from Europe, Asia, Australia, and New Zealand including 832 patients on DOACs and 32 375 patients not on anticoagulants, building on a previous European initiative [24, 39]. This large multinational study showed a lower risk of sICH in patients treated with DOACs (adjusted OR 0.57; 95% CI 0.36 to 0.92) [24]. Adjusted data (age, hypertension, baseline NIHSS score, premorbid functional independence, admission blood pressure, glucose plasma level) indicated no increased risk of sICH associated with recent DOAC use (within 48 h) before IVT, irrespective of DOAC plasma levels or use of idarucizumab. The lower risk of sICH (2.5%; 95% CI 1.6 to 3.8) vs. 4.1%; 95% CI 3.9 to 4.4) was true for all selection strategies (no restrictions, reversal or activity measurement). One explanation for this could be selection bias. Another explanation is that DOAC treatment leads to smaller infarcts with a lower risk of hemorrhagic transformation. However, there could also be a more basic pathophysiologic explanation for this result [12]. As described above, no increased or even a lower risk of bleeding was found in animal models [28–33]. For example, thrombin inhibition may minimize the disruption of the blood–brain barrier and lead to less tissue damage or make the brain less susceptible to hemorrhagic transformation [12].

In Switzerland, liberalization of local guidelines for IVT regardless of recent DOAC intake showed no safety concerns in a single-center target trial analysis [40]. Among 98 consecutive DOAC patients eligible for IVT, 49 received IVT and 49 did not. No sICH occurred in those receiving IVT, compared to 2 out of 49 patients not receiving IVT (adjusted difference − 2.5%; 95% CI − 5.9 to 0.8). Rates of any radiologic ICH were similar between the groups.

A meta-analysis of 14 retrospective cohort studies compared the risk of ICH after IVT in patients on DOACs to those not on anticoagulants before their ischemic stroke [41]. The pooled analyses showed no difference in the rates of sICH (3.4% vs. 3.5%; OR 0.95; 95% CI 0.67 to 1.36) or any intracranial hemorrhage (17.7% vs. 17.3%; OR 1.23; 95% CI 0.61 to 2.48) between the DOAC group and those not on DOAC. Three of the 14 studies included in the meta-analysis counted for 84.7% of the total weight [24, 37, 38].

To sum up, all these retrospective studies (Table 1) point in the same direction, namely that IVT seems as safe in patients with a recent DOAC intake as in patients not on oral anticoagulation. It should be noted that we only have retrospective data on selected patients, and the numbers treated in most of these cohorts are small. Overall, 160 of 3983 (4.0%) patients with a recent intake of DOAC treated with IVT-experienced sICH [24, 34–40, 42]. These nine cohorts have substantial variations in their definitions of recent DOAC intake and of sICH. Further, there may be a small overlap between some of the cohorts, and among those treated, there is probably a selection bias toward a lower risk of sICH. A systematic review and meta-analysis including six of these cohorts showed significant methodological heterogeneity, selection bias and missing clinical data that further limit the validity of the results [41]. Thus, based on the existing knowledge it is only reasonable to say that IVT seems safe in selected patients with a recent intake of DOAC. Noteworthy, the safety data is still better than the data available before IVT was recommend in patients on VKA with INR ≤ 1.7 [43]. Based on the above-mentioned limitations of all the included studies, there exist no evidence of the efficacy of IVT in patients with a recent intake of DOAC.

## Evidence from other direct anticoagulants

Argatroban is an intravenous thrombin inhibitor commonly used during interventional procedures in cardiology, licensed back in 2000. Recently argatroban was tested in a Chinese multicenter, open-label, randomized controlled trial (ARAIS) [44]. This study included 817 patients qualifying for IVT: 402 received argatroban (bolus followed by 48-h infusion) plus alteplase, and 415 received alteplase alone. The rates of sICH were similar between the two groups. The MOST study compared a similar approach with argatroban given within 75 min of thrombolysis, but with even higher doses of argatroban than ARAIS [45]. The MOST study was stopped for futility in the summer for 2023 after a pre-planned analysis of the first 500 patients showed no statistically significant signal of harm in the high-dose argatroban arm that enrolled 59 patients [46]. However, argatroban is not fully comparable to DOAC treatment which mostly consists of factor Xa antagonists so these studies cannot be used to imply that IVT in patients with DOAC intake is safe. In addition, patients included in randomized controlled trials are usually less fragile than real world patients.

## Anticoagulant activity measurements prior to IVT

Most guidelines include the option to use IVT in patients on DOAC if specific coagulation tests indicate safety [6, 7].

The International Committee for Standardisation in Haematology and the International Committee on Thrombosis and Haemostasis emphasize the following points regarding anticoagulant activity measurements in DOAC: (1) prothrombin time/INR and APTT are unreliable, (2) current non-specific point-of-care tests fail to adequately detect DOACs, and (3) while tandem mass spectrometry is the gold standard for measuring DOACs, drug-calibrated tests like dilute thrombin time, ecarin chromogenic assay, ecarin clotting time, anti-Factor IIa, and anti-Factor Xa are suitable for rapid quantification [47–49].

Ecarin clotting time (for dabigatran) and calibrated anti-Xa assays (for Xa inhibitors) seems to display linear, dose-dependent correlations with serum levels [50].

Challenges in the use of specific coagulation tests in clinical practice include limited availability, no established clear cut-off values, biologic variability, and test inconsistencies. Further, the timepoint of the last intake must be known if a plasma-level measurement should be interpreted correctly. New approaches, including urine dipstick tests, are under investigation, but will probably have at least some of the same challenges as today’s available tests [47, 48, 51].

Acute stroke is an emergency situation, and long turnaround times of tests should not delay the decision regarding IVT. The turnaround time for specific assays is improving, real-world data from a recent Norwegian study had a median turnaround time for the drug measurements of 38 (interquartile range 33–46) min [52]. Among 148 patients with a recent intake of DOAC and symptoms of acute ischemic stroke, only four patients were given IVT based on the test results using a very low cut-off of acceptable DOAC plasma concentrations. The results of this study highlight the difficulties in feasibility and clinical utility of the available tests to aid IVT decisions in the emergency room given the missing established cut-offs.

## Reversal agent use prior to IVT

There are currently two commercially available reversal agents for DOACs: andexanet alfa and idarucizumab. Andexanet alfa is a recombinant human Factor Xa variant that imitates native Xa [53]. Idarucizumab is a humanized monoclonal antibody fragment with a high affinity for dabigatran [54]. Both agents have demonstrated dose-dependent reversal of DOAC activity in phase 3 trials [53]. Observational studies including patients with major bleeding have shown that normal drug-specific activity levels can be restored within min [53]. Idarucizumab is approved for dabigatran reversal including urgent procedures, and andexanet alfa recently received approval for reversing rivaroxaban and apixaban (though not yet for edoxaban) when reversal is needed due to life-threatening or uncontrolled bleeding.

Several case-series, case–control and observational studies support the use of idarucizumab before IVT in patients with recent intake of dabigatran [55–61]. European expert opinion recommended idarucizumab prior to IVT [6]. Noteworthy, there exists no randomized trials of idarucizumab vs. no idarucizumab prior to IVT in patients with a recent intake of dabigatran. Idarucizumab is expensive, and the cost-effectiveness of reversal approaches in IVT remains uncertain.

Very few cases of reversal with Andexanet alfa prior to IVT are published and European expert opinion recommends against its use before IVT given its associated signal of thrombotic complications [6]. In addition to reported thromboembolic complications, the logistics of administration require a bolus followed by a 2-h infusion with a potential Xa-activity rebound, complicating its use in a time sensitive condition like ischemic stroke.

## Evidence of MT in patients with recent intake of DOAC

Data regarding safety and efficacy of MT for large vessel occlusion in patients with a recent intake of DOAC are based on observational studies [26, 27, 62–65].

A French multicenter prospective database compared MT in patients with a large vessel occlusion that were on DOAC or VKA [63]. Inclusion criteria were last intake within 24 h and no IVT prior to MT. Data on safety outcomes were numerical, but not statistically, in favor of DOAC with sICH present in 5.7% (6/105) of DOAC patients and in 12.4% (12/97) of those treated with VKA. Clinical outcome appeared to be better with DOAC than with VKA, but missing data and potential confounding mean that these data should be interpreted with caution. Further, the study did not compare patients with a recent intake of DOAC with patients not on DOAC.

Data from 25 MT centers in Germany also suggest that MT in patients on DOAC is safe [27]. This study compared 827 patients with MT and a recent intake of DOAC with 4867 patients without oral anticoagulation. In the no-DOAC group, 59.4% (2892/4867) received IVT, while the corresponding figure in the DOAC group was 11.0% (91/827). Adjusted analysis revealed no statistically significant difference in safety between DOAC use and no anticoagulation (OR 0.90; 95% CI 0.67 to 1.20). The efficacy of MT was similar in the two groups. This study did not present a clear definition of ICH, the definition of recent DOAC intake was based on the local physician’s interpretation, no information was given about reversal, and no DOAC levels were measured. In addition, there was a surprisingly high proportion of patients on DOAC that received IVT prior to MT making the generalisability difficult.

A subgroup analysis of the MR CLEAN registry found no increased risk of sICH in patients with prior oral anticoagulation compared to those without oral anticoagulation [62].

Before MT, 2660 patients were not using anticoagulation while 502 used oral anticoagulation (DOAC *n* = 98, VKA *n* = 404). The incidence of sICH was numerically lower in patients on DOACs (1/98, 1%) when compared with patients on VKAs (23/404, 5.7%) or without any oral anticoagulation (162/2660, 6.1%). However, the numbers of patients on DOAC make it hard to perform any meaningful statistical subgroup analysis. Further, no definition of recent anticoagulation intake is given.

A Swiss study from the BEYOND- SWIFT database compared 1612 patients with no anticoagulation with 222 patients on VKA and 98 patients on DOAC [26]. Five out of 98 (5.2%) patients on DOAC had sICH which was similar to those without anticoagulation (84/1622, 5.2%). VKA was associated with increased odds for sICH (aOR 2.55; 95% CI 1.35 to 4.84) as compared with the control group, whereas no association with DOAC intake was observed (aOR 0.98; 95% CI 0.29 to 3.35). This study also included a meta-analysis with 7462 patients (855 VKAs, 318 DOACs, and 6289 controls) from 15 observational cohorts. This 2020 meta-analysis concluded that patients on VKA (aOR 1.62; 95% CI 1.22 to 2.17), but not patients on DOAC (aOR 1.03; 95% CI 0.60 to 1.80), had an increased risk of sICH after MT as compared with control patients without anticoagulation [26].

MT of occlusions in the medium and distal vessels of the middle cerebral artery (M2, M3, and M4 segments) in anticoagulated patients have been evaluated in a retrospective analysis involving 1282 patients from 37 centers across North America, Asia, and Europe [66]. This study only included patients who underwent MT without IVT. The definition of anticoagulated included patients who were on VKAs or DOACs upon presentation. In total, 223 (34%) were on anticoagulation therapy. There were no differences in functional or safety outcomes between the group anticoagulated and those without anticoagulation. Unfortunately, this study did not distinguish type of oral anticoagulation (DOACs or VKAs), the definition of recent intake of anticoagulation was quite broad, and there is probably an inherited selection bias and potential weakness by studying a group of patients that were offered MT for medium and distal vessels before this procedure had an evidence-based [66].

Although no randomized controlled trials exist, all the available literature points in one direction, namely that MT for large vessel occlusion is reasonably safe in patients with a recent intake of DOAC. The decision to proceed with MT in a patient who has recently taken a DOAC should be made after weighing the risks and benefits including individual patient factors, type of occlusion and the stroke severity. Some contradicting findings of whether MT is more effective in DOAC patients than in non-DOAC patients exist. Due to the heterogeneity of the DOAC and MT studies, it is not possible to establish any evidence for whether this is a reflection of varied clinical practice or a true biologic effect of DOAC.

## Suggestion for management until better evidence is available

**Fig. 1:**
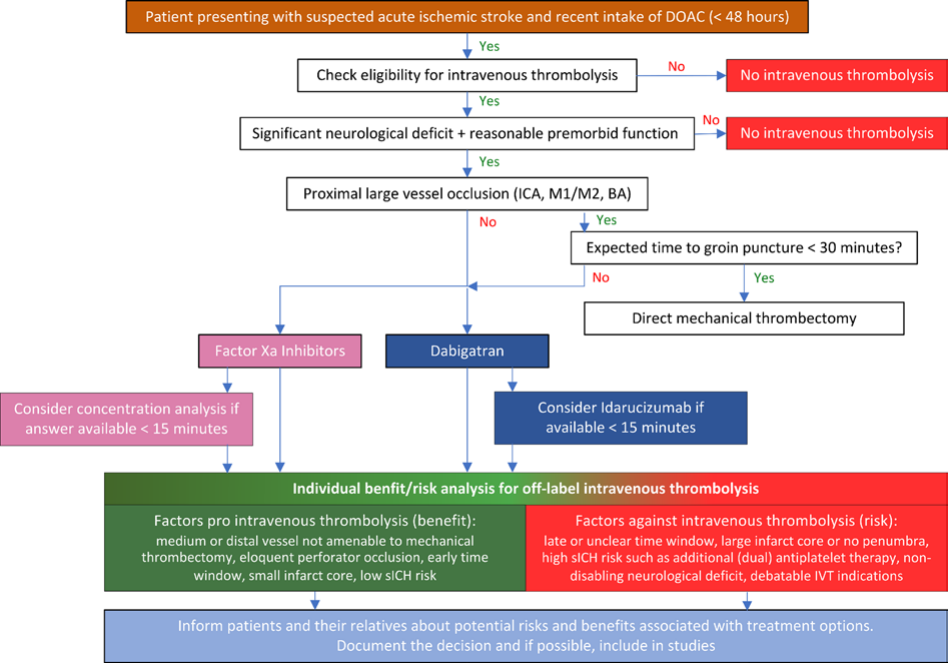
Our expert opinion individualized risk–benefit assessment for managing patients with acute ischemic stroke who have recently taken direct oral anticoagulants (DOACs). IVT = intravenous thrombolysis; ICA: internal carotid artery; M1: first segment of middle cerebral artery; M2: second segment of middle cerebral artery; BA: basilar artery; DOAC: direct oral anticoagulant; sICH: symptomatic intracranial hemorrhage

Controversy surrounds the safety of IVT in acute ischemic stroke patients treated with DOAC. Thus, we suggest an individualized risk–benefit approach in off-label use of IVT in patients with last intake of DOAC < 48 h. The aim of all acute recanalization therapies is always to increase the chance of a good functional outcome, and to decrease the risk of sICH and poor functional outcome. Since sICH is a devastating and often lethal complication when occurring in stroke patients treated with IVT, careful considerations must be taken [9, 67]. A wide range of risk factors for sICH have been reported such as older age, higher NIHSS, atrial fibrillation, large vessel occlusion, diabetes, hyperglycemia, uncontrolled hypertension, combination antiplatelet use, large areas of early ischemic change and treatment in the later part of the 4.5-h time window [9–11, 68, 69]. Many of these risk factors are found in a significant proportion of patients with acute ischemic stroke, and it is hard to extrapolate the results to each individual potentially eligible for IVT. Further, many of the results of risk factors of sICH have been contradictory, and they have mainly been identified in the secondary analysis of randomized controlled trials or retrospective studies. Taken together with the fact that DOAC patients tend to be fragile with more severe comorbidities and polypharmacy than stroke patients not using DOACs, there is a need for an individual assessment of the patient prior to IVT. Thus, we recommend an individualized risk–benefit assessment for managing patients with acute ischemic stroke who have recently taken DOACs (Fig. 1). This approach involves:

- **1. Evaluating IVT eligibility**: Assess whether the patient otherwise meets criteria for IVT, has a significant neurologic deficit, and the risk of sICH based on established prediction models.
- **2. Considering alternative treatments**: Explore the availability of alternative interventions like MT if a proximal large vessel occlusion is present as well as the expected delay to MT.
- **3. Utilizing reversal agents and coagulation tests**: If immediate MT is not an option, consider using reversal agents and appropriate coagulation tests to manage anticoagulation status. However, remember that acute stroke treatment is time-dependent and that safe cut-off levels are not established. Furthermore, a plasma-level measurement on admission can only be interpreted correctly if the timepoint of the last dose is known since the levels might still be undetectable early after intake. Consider to consult expert hematology input regarding these issues
- **4. Consulting with stroke specialists**: Involve experienced stroke physicians in the decision-making process to ensure that expert opinions are incorporated
- **5. Patient and family communication**: Clearly inform patients or their relatives about the potential risks and benefits associated with the proposed treatments
- **6. Documentation**: Document all off-label decisions and discussions thoroughly to ensure clarity and accountability

By following this structured approach, clinicians can make informed, patient-centered decisions that balance the risks and benefits of the different treatment options for patients presenting with acute ischemic stroke and a recent intake of DOAC.

## Conclusions and future directions

To sum up, DOAC is the most frequent absolute contraindication for IVT. The inclusion of recent DOAC intake as an absolute contraindication for IVT is based on the presumption of an increased risk of sICH, but there is lack of evidence for this recommendation. The number of patients with DOAC therapy will increase considerably, these patients tend to be more fragile and have more severe strokes, and the clinical problems of what to do in the acute setting with these patients will be even more commonplace in the coming years. So far, we only have retrospective data on selected patients, and the number treated in each cohort is small. We lack prospective studies and randomized controlled trials. Future studies will hopefully contribute to better prediction models (including clinical and imaging markers) with a more personalized approach with regards to risk for intracerebral hemorrhage after IVT. However, today’s safety data for DOAC is still better than the data available before IVT was recommended in patients on VKA with an INR ≤ 1.7 [43]. There are no safety signals of MT in patients with a recent DOAC intake so MT is considered safe for those with a large vessel occlusion.

Several international efforts are under way to provide robust evidence and hopefully fill the knowledge gap regarding safety and efficacy of IVT in patients on DOAC. The largest one is the global DOAC Intravenous Thrombolysis (DO-IT) study (NCT06571149). This is a prospective, randomized controlled trial involving about 100 centers across 15 countries that will include approximately 900 patients. The DO-IT study will also be accompanied by a prospective registry study, which will capture all consecutive acute ischemic stroke patients with recent DOAC intake (NCT06556446). In addition, the Safe IVT FXa trial and the ACT-GLOBAL adaptive platform trial, will include patients with recent intake of DOAC and IVT.

Until the results of these trials are known, we suggest that IVT in patients with a recent intake of DOAC needs an individualized risk–benefit assessment. In short, if there is a large vessel occlusion, then the patients should be directed to MT if available immediately. If the stroke is caused by an eloquent distal or perforator occlusion, and the patient has significant neurologic deficit, short thrombus, and a low risk of sICH—consider off-label use of IVT for these patients.

## Data Availability

Not applicable for this kind of review.
